# Impact of silymarin‐supplemented cookies on liver enzyme and inflammatory markers in non‐alcoholic fatty liver disease patients

**DOI:** 10.1002/fsn3.4348

**Published:** 2024-07-17

**Authors:** Hafiza Madiha Jaffar, Huma Bader ul Ain, Tabussam Tufail, Asif Hanif, Tabarak Malik

**Affiliations:** ^1^ Faculty of Allied Health Sciences University Institute of Diet & Nutritional Sciences, University of Lahore Lahore Pakistan; ^2^ School of Food and Biological Engineering Jiangsu University Zhenjiang China; ^3^ Allied Health Sciences The University of Lahore Lahore Pakistan; ^4^ Department of Biomedical Sciences Jimma University Jimma Ethiopia; ^5^ Present address: Division of Research & Development Lovely Professional University Phagwara Punjab 144001 India

**Keywords:** cookies, inflammatory markers, lipid profiles, liver enzymes, NAFLD, silymarin

## Abstract

Nonalcoholic fatty liver disease (NAFLD) is a growing public health concern characterized by fat accumulation and severe disorders like nonalcoholic steatohepatitis (NASH), which are influenced by obesity, inflammatory processes, and metabolic pathways. This research investigates the potential of silymarin‐supplemented cookies in managing NAFLD by evaluating their impact on liver enzyme activity, inflammatory markers, and lipid profiles. A clinical trial in Lahore, Pakistan, involved 64 NAFLD patients. Participants were divided into placebo and three treatment groups, with the latter receiving silymarin‐supplemented cookies for 3 months. The study assessed liver enzyme levels and inflammatory markers, at baseline and after the intervention, utilizing statistical analyses to evaluate differences. The lipid profile and renal function test (RFT) were also measured at baseline and after 3 months in each group for safety assessment. After 3 months, the treatment groups indicated more significant decreases in liver enzymes compared to the placebo group (*p* ≤ .05). Treatment 3 showed significant reductions in alanine aminotransferase (ALT) (64.39–49.38 U/L) and aspartate aminotransferase (AST) (61.53–45.38 U/L). Treatment 3 also showed improvements in alkaline phosphatase (ALP) levels and the AST/ALT ratio. Additionally, the treatment group demonstrated a significant reduction in inflammatory markers. Treatment 3 showed a significant decrease in C‐reactive protein (CRP) (6.32–3.39 mg/L) and erythrocyte sedimentation rate (ESR) (38.72–23.86 mm/h), indicating that individuals with NAFLD may benefit from the intervention's potential benefits in lowering inflammation. The study revealed that an intervention significantly improved the inflammatory markers, liver enzymes, and lipid profiles of NAFLD participants, suggesting potential benefits for liver health.

## Graphical abstract

The study investigates the potential of silymarin‐supplemented cookies in managing nonalcoholic fatty liver disease (NAFLD), a growing public health concern characterized by fat accumulation and severe disorders like nonalcoholic steatohepatitis (NASH). A clinical trial in Lahore, Pakistan, involved 64 NAFLD patients divided into placebo and three treatment groups. After 3 months, the treatment groups showed significant decreases in liver enzymes, alanine aminotransferase (ALT) and aspartate aminotransferase (AST) levels, alkaline phosphatase (ALP) levels, and AST/ALT ratio. Additionally, the treatment group showed a significant reduction in inflammatory markers, C‐reactive protein (CRP) and erythrocyte sedimentation rate (ESR). The study concluded that the intervention significantly improved the inflammatory markers, liver enzymes, and lipid profiles of NAFLD participants, suggesting potential benefits for liver health.

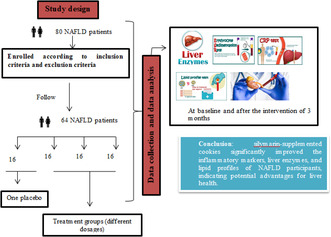

## 1 INTRODUCTION

Nonalcoholic fatty liver disease (NAFLD) is a growing public health concern due to its increased prevalence compared to other chronic liver diseases. A group of liver diseases known as NAFLD are typified by lipid buildup, which damages the liver and raises enzyme levels (Kumar et al., 2020; Lazarus et al., 2022; Pafili & Roden, 2021). Insulin resistance, sedentary lifestyles, high‐fat diets, obesity, and hepatic lipid buildup are among the metabolic and inflammatory processes that lead to NAFLD. The formation of reactive oxygen species (ROS), lipotoxicity, oxidative stress, elevated lipid peroxidation, and mitochondrial dysfunction are all components of the inflammatory pathways (Chen et al., 2020; Di Ciaula et al., 2021; Ullah et al., 2019; Ziolkowska et al., 2021). The World Gastroenterology Organization (WGO) recommends a tiered approach for diagnosing NAFLD, prioritizing noninvasive methods like ultrasonography, serum biomarkers, and liver enzyme assessments over expensive procedures like liver biopsies (Castagneto‐Gissey et al., 2023; Forlano et al., 2023). Common liver injury markers include alanine aminotransferase (ALT), aspartate aminotransferase (AST), alkaline phosphatase (ALP), y‐glutamyltransferase (GGT), and bilirubin. Studies show correlations between serum biomarkers and NAFLD severity. Currently, no specific pharmaceutical treatments are approved for NAFLD (Bagherniya et al., 2018; Fu et al., 2020; Tamber et al., 2023).

In NAFLD patients, metabolic comorbidities, such as obesity, dyslipidemias, diabetes, and insulin resistance, should be managed with dietary adjustments, increased physical activity, and weight control. However, many people find it challenging to maintain these benefits once they get going. Natural supplements with hepatoprotective and antioxidant properties may be helpful to manage NAFLD and stop the condition from getting worse and developing into fibrosis and nonalcoholic steatohepatitis (NASH) (Pouwels et al., 2022; Ratziu et al., 2019; Younossi et al., 2021, 2023). Milk thistle, also known as *Silybum marianum* (Marceddu et al., 2022), has the potential to alleviate NAFLD symptoms by stimulating cellular glutathione content, enhancing membrane stability, regulating nuclear expression, and inhibiting the transformation of stellate hepatocytes into myofibroblasts, thus mitigating fibrosis and tissue degeneration in severe hepatic diseases (Hong et al., 2015; Nawaz et al., 2023; Samee et al., 2023; Zahran, 2015). Research has demonstrated that silymarin can improve liver enzymes, modify inflammatory pathways, and improve the general health status of people with NAFLD when used in conjunction with other antioxidant and anti‐inflammatory supplements, such as vitamin E, vitamin C, coenzyme Q10, and selenium (Aghemo et al., 2022; Akhtar et al., 2023; Camini & Costa, 2020; Surai et al., 2024; Zhang et al., 2023). Silymarin has also been shown to ameliorate blood parameters, alleviate symptoms, and improve ultrasonography results in NAFLD patients (Colletta et al., 2020; Hüttl et al., 2021; Surai et al., 2024).

The purpose of this study was to assess the impact of silymarin‐supplemented cookies on inflammatory indicators, lipid profiles, and liver enzyme activity in individuals with NAFLD. The analysis of these markers will provide valuable insights into the potential of these cookie supplements to benefit individuals with NAFLD and advance our understanding of how modified silymarin supplementation may modulate liver function and inflammation in this patient population.

## 2 MATERIALS AND METHODS

The NAFLD patients in this study participated in a randomized controlled clinical trial that spanned from August to October 2023. The current trial was conducted on 64 patients at Sir Ganga Ram Hospital in Lahore, Pakistan, with participants selected based on factors like history, physical examination, laboratory evidence, age, medical history, and biochemical parameters.

Inclusion criteria encompassed specific health indicators, age range, recent medical history, and specific biochemical thresholds, including age between 25 and 40 years, males and females, recent medical history (<1 month), and elevated ALT and AST levels (ranges: 7–56 and 5–40 U/L, respectively), which were meticulously screened and enrolled. Exclusion criteria included other causes of fatty liver and abnormal liver function, cirrhosis, excessive alcohol consumption, pregnancy or breastfeeding, advanced liver disease, underlying liver diseases, diabetes, metabolic syndromes, or prior treatment with other medications.

### 2.1 Sample size

The study involved 64 participants, with 16 in each group, to achieve a power of 99% at a 5% significance level. With a 20% dropout rate, the final sample size was 80, with 20 in each group. The departmental Ethical Committee approved the study protocol, and all patients provided written informed consent.

### 2.2 Study design

The investigation utilized specific equipment and a treatment plan, including a Seca 206 wall meter for height measurement, a Beurer digital GS34 bargraph for weight measurement, and Pars Azmoon kits for liver enzyme assessment. The 3‐month therapy protocol consisted of oral silymarin‐supplemented cookies, with participants divided into four groups (one placebo and three treatment), as indicated by Table 1.

**TABLE 1:** Treatments to be studied.

Treatment groups	Intervention	Dose of cookies/day	Duration/days
Placebo group (T_0_)	Simple cookies	2 (1 morning and 1 evening)	90
Treatment group 1 (T_1_)	100 mg silymarin‐supplemented cookies	2 (1 morning and 1 evening)	90
Treatment group (T_2_)	100 mg silymarin‐supplemented cookies	3 (1 ½ in morning and 1 ½ in evening)	90
Treatment group 3 (T_3_)	100 mg silymarin‐supplemented cookies	4 (2 in morning and 2 in evening)	90

### 2.3 Medical assessments

The study involved randomly assigning subjects to placebo or experimental groups, calculating body mass index (BMI), and conducting laboratory evaluations. Participants followed a daily treatment (cookie) schedule for 3 months, with biochemical testing, anthropometric measurements, and medical history evaluation. Biochemical markers were measured at the start and end of the investigation.

### 2.4 Statistical evaluation

The statistical analysis was carried out using the Macintosh version 20.0 of the SPSS (Statistical Package for Social Sciences, Chicago, IL, USA) program. The means and standard deviation were used to express the results along with median (Min–Max). Using the Kolmogorov–Smirnov or Shapiro–Wilk test, the normality of the variables was determined. The Kruskal–Wallis test (for non‐normally distributed data) and one‐way analysis of variance (ANOVA) (for regularly distributed variables) were used to identify statistically significant differences between group comparisons. Analysis that had a *p*‐value less than .05 was considered significant.

## 3 RESULTS

**FIGURE 1:**
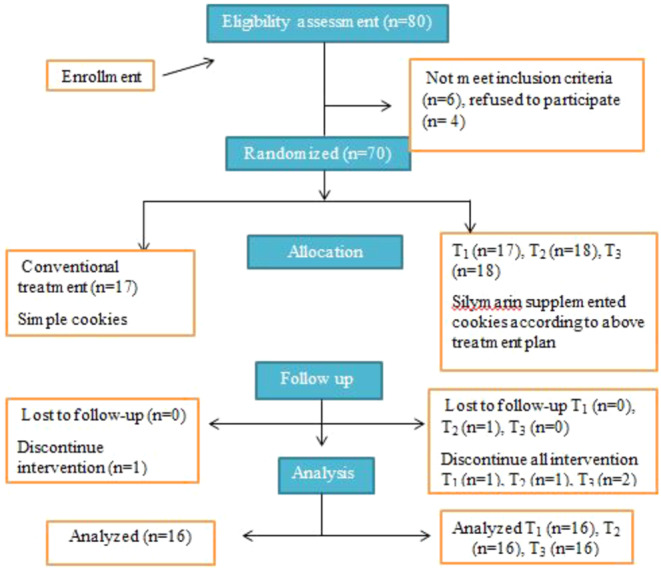
Flowchart of participant enrollments.

The consort diagram of the current study is shown in Figure 1. During the study period, 80 NAFLD patients were screened for recruitment, of whom 70 were included in this trial: 17 in the placebo group, 17 in the first treatment group, 18 in the second treatment group, and 18 in the third treatment group. Two days after the initiation of interventions, one participant declined to continue on the placebo, one from the first treatment group, one from the second treatment group, and two from the third treatment group, while one patient was excluded due to nonregular use of treatment (lost follow‐up) from the second treatment group. So finally, 64 patients completed the current study (Figure 1), including 16 in each group.

The patient's qualitative and quantitative demographic data are presented in Table 2. The table presents baseline characteristics of participants, including age distribution, height, weight, gender, marital status, education, severity of disease, duration of disease, and the presence of sleep disturbance issues across all groups (Placebo ‐T0, Treatment 1 ‐T1, Treatment 2 ‐T2, and Treatment 3 ‐T3). Mean values with standard deviations, or percentages, are provided for each variable, offering a comprehensive overview of the study population and facilitating comparisons between the different treatment groups.

**TABLE 2:** The frequency distribution of participant's demographic qualitative and quantitative variables at baseline of all studied groups.

Variables	T_0_	T_1_	T_2_	T_3_
	Mean ± SD or *n* (%)	Mean ± SD or *n* (%)	Mean ± SD or *n* (%)	Mean ± SD or *n* (%)
Age (years)				
25–30	4 (25)	5 (31.25)	5 (31.25)	3 (18.75)
31–35	9 (56.25)	10 (62.5)	11 (68.75)	12 (75)
36–40	3 (18.75)	1 (6.25)	0 (0)	1 (6.25)
Height, cm	167.56 ± 10.48	165.64 ± 10.06	164.59 ± 9.92	167.33 ± 10.27
Weight, kg	67.97 ± 12.64	68.72 ± 12.46	67.54 ± 16.02	69.22 ± 10.59
Gender				
Male	8 (50)	7 (43.75)	6 (37.5)	8 (50)
Female	8 (50)	9 (56.25)	10 (62.5)	8 (50)
Marital status				
Single	5 (31.25)	2 (12.5)	5 (31.25)	3 (18.75)
Married	11 (68.75)	14 (87.5)	11 (68.75)	13 (81.25)
Education				
Under matric	5 (31.25)	5 (31.25)	7 (43.75)	4 (25)
Bachelor's degree	7 (43.75)	7 (43.75)	4 (25)	9 (56.25)
Masters	3 (18.75)	4 (25)	3 (18.75)	2 (12.5)
Ph.D.	1 (6.25)	0 (0)	2 (12.5)	1 (6.25)
Severity of disease				
Mild	9 (56.25)	10 (62.5)	11 (68.75)	8 (50)
Moderate	7 (43.75)	6 (37.5)	5 (31.25)	8 (50)
Duration of disease				
Years	5.75 ± 5.35	4.37 ± 3.03	4.26 ± 2.83	5.4 ± 4.5
Sleep disturbance issue				
Yes	5 (31.25)	10 (62.5)	2 (12.5)	7 (43.75)
No	11 (68.75)	6 (37.5)	14 (87.5)	9 (56.25)

*Note*: All values are means ± SDs; *n* = total patients in every group. Placebo‐ simple cookies, T^1^‐2 (1 morning and 1 evening), T^2^‐3 (1 ½ in morning and 1 ½ in evening), T^4^‐4 (2 in morning and 2 in evening).

**FIGURE 2:**
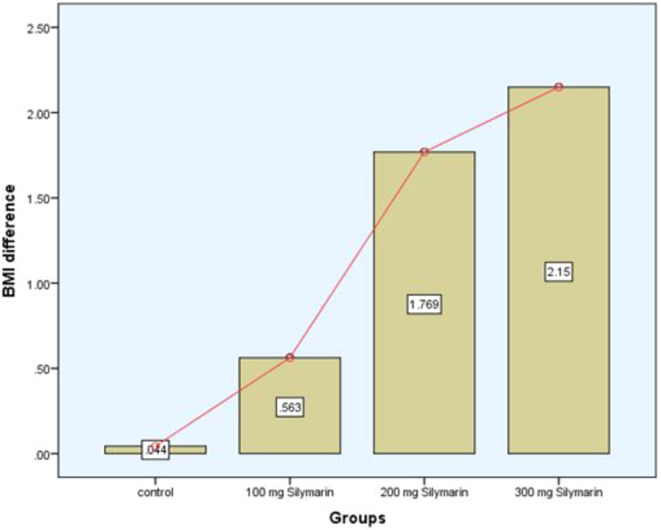
Body mass index (BMI) difference of all group participants between baseline and after 3 months.

Table 3 presents an in‐depth analysis of the treatment effects of silymarin‐supplemented cookies at varying dosages (low: 200 mg, medium: 300 mg, and high: 400 mg), as well as baseline and 3‐month body mass index (BMI) measurements in individuals with NAFLD. The BMI of the placebo group changed very little, whereas Treatment 3 showed a significant drop (*p*‐value = .000) from 27.25 to 25.10 kg/m^2^, suggesting that increased silymarin concentrations may have an effect on body weight. Figure 2 shows the difference between baseline and after 3 months of the study. Notably, the observed differences are validated by the statistical analyses (ANOVA for parametric distribution because the data were normal). All of these results point to the possibility that silymarin‐supplemented cookies, especially at higher concentrations, can help people with NAFLD achieve a lower body mass index. Therefore, in order to corroborate these results and clarify the underlying processes of silymarin's possible therapeutic advantages as indicated in a previous study (Fallah et al., 2021), Silymarin (milk thistle extract) serves as a therapeutic agent in gastrointestinal cancer.

**TABLE 3:** Body mass index (BMI) of all group participants with NAFLD at baseline and after 3 months.

Groups	Parameters	
	BMI (kg/m^2^)	BMI (kg/m^2^)
	Before	After
Placebo		
Mean ± SD	27.38 ± 1.25	27.34 ± 1.16
Median (Min–Max)	27.25 (25.90–29.80)	27.35 (25.50–29.70)
Treatment 1		
Mean ± SD	27.10 ± 0.90	26.53 ± 0.86
Median (Min–Max)	27.30 (25.70–28.70)	26.75 (24.20–27.80)
Treatment 2		
Mean ± SD	28.13 ± 1.35	26.36 ± 1.26
Median (Min–Max)	28.25 (25.70–30.00)	26.50 (24.20–28.30)
Treatment 3		
Mean ± SD	27.25 ± 1.39	25.10 ± 0.82
Median (Min–Max)	27.30 (25.30–29.40)	25.20 (24.00–26.30)
*p*‐Value^a^, ^b^	.99	.000

*Note*: Placebo‐ simple cookies, T^1^‐2 (1 morning and 1 evening), T^2^‐3 (1 ½ in morning and 1 ½ in evening), T^4^‐4 (2 in morning and 2 in evening).

Abbreviations: BMI, body mass index; NAFLD, nonalcoholic fatty liver disease.

^a^ Kruskal–Wallis test.

^b^ One‐way ANOVA.

**FIGURE 3:**
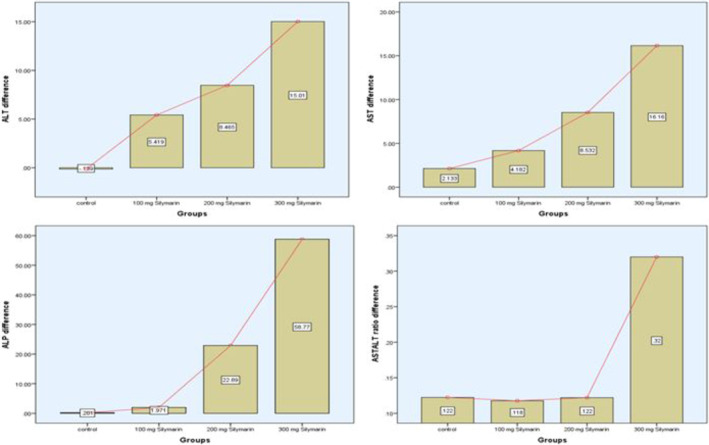
Difference of liver enzymes of all group participants between baseline and after 3 months.

Table 4 provides a detailed analysis of the liver enzyme levels in patients diagnosed with NAFLD, both at baseline and 3 months after consuming cookies enhanced with silymarin at several dosages (low: 200 mg, medium: 300 mg, and high: 400 mg). The placebo group's aspartate aminotransferase (AST) and alanine aminotransferase (ALT) levels exhibited relatively modest fluctuations, with mean values remaining mostly constant. On the other hand, ALT levels significantly decreased in Treatment 3 (with the greatest concentration of silymarin) from 64.39 to 49.38 U/L (*p*‐value = .000), indicating a possible hepatoprotective impact. Comparably, AST levels dropped dramatically (*p*‐value = .000) from 61.53 to 45.38 U/L, suggesting a beneficial effect on liver function. Treatment 2 similarly showed significant reductions in ALT and AST levels while having medium silymarin content. Treatment 3 resulted in a statistically significant decrease in alkaline phosphatase (ALP) levels (*p*‐value = .001), highlighting the possible contribution of increased silymarin concentrations to the mitigation of liver enzyme abnormalities linked to NAFLD. Treatment 3 resulted in a significant improvement in the AST/ALT ratio (*p*‐value = .000), indicating improved liver function. Figure 3 shows the difference between baseline and after 3 months of the study.

**TABLE 4:** Liver enzymes of all group participants with NAFLD at baseline and after 3 months.

Groups	Parameters (U/L)							
	ALT	ALT	AST	AST	ALP	ALP	AST/ALT ratio	AST/ALT ratio
	Before	After	Before	After	Before	After	Before	After
Placebo								
Mean ± SD	61.83 ± 4.88	61.97 ± 4.63	64.58 ± 4.16	62.44 ± 5.66	241.31 ± 5.87	241.10 ± 6.04	1.01 ± 0.042	0.89 ± 0.036
Median (Min–Max)	61.24 (53.14–70.77)	61.46 (53.42–69.66)	63.72 (59.48–71.50)	60.79 (53.42–71.91)	240.92 (231.83–251.45)	240.83 (230.60–250.37)	1.01 (0.95–1.09)	0.89 (0.84–0.96)
Treatment 1								
Mean ± SD	61.57 ± 3.58	56.15 ± 3.99	60.93 ± 6.07	56.75 ± 5.09	239.35 ± 5.68	237.38 ± 4.83	0.99 ± 0.034	0.87 ± 0.030
Median (Min–Max)	61.52 (55.99–70.53)	55.66 (49.28–62.28)	59.09 (53.12–72.08)	56.46 (50.03–66.11)	239.16 (230.88–250.76)	236.76 (230.76–247.98)	0.98 (0.95–1.08)	0.87 (0.84–0.95)
Treatment 2								
Mean ± SD	62.96 ± 4.47	54.49 ± 5.37	63.70 ± 4.86	55.16 ± 3.86	239.83 ± 5.69	216.94 ± 16.01	1.00 ± 0.038	0.88 ± 0.029
Median (Min–Max)	63.40 (54.53–71.90)	54.00 (48.49–66.20)	63.38 (54.25–72.10)	55.27 (50.03–62.50)	238.30 (232.21–250.84)	222.05 (195.25–240.32)	1.01 (0.94–1.08)	0.88 (0.83–0.95)
Treatment 3								
Mean ± SD	64.39 ± 4.28	49.38 ± 2.07	61.53 ± 6.10	45.38 ± 5.29	240.84 ± 4.82	182.07 ± 16.16	0.97 ± 0.063	0.65 ± 0.068
Median (Min–Max)	64.97 (55.37–71.34)	49.44 (45.72–52.70)	60.59 (53.42–71.34)	45.01 (38.71–55.91)	239.05 (234.58–250.13)	181.95 (158.41–209.31)	0.98 (0.86–1.08)	0.64 (0.55–0.84)
*p*‐value	.250^b^	.000^b^	.183^b^	.000^b^	.739^b^	.000^a^	.080^b^	.000^b^

*Note*: Placebo‐ simple cookies, T^1^‐2 (1 morning and 1 evening), T^2^‐3 (1 ½ in morning and 1 ½ in evening), T^4^‐4 (2 in morning and 2 in evening).

Abbreviations: ALT, alanine aminotransferase; AST, aspartate aminotransferase; ALP, alkaline phosphatase; NAFLD, nonalcoholic fatty liver disease.

^a^ Kruskal–Wallis test.

^b^ One‐way ANOVA.

All of these findings highlight the possible hepatoprotective benefits of cookies containing silymarin, with Treatment 3 showing the best results. The statistical studies, such as the Kruskal–Wallis tests and ANOVA, confirm the observed differences and clarify the underlying mechanisms of silymarin in the treatment of NAFLD.

**FIGURE 4:**
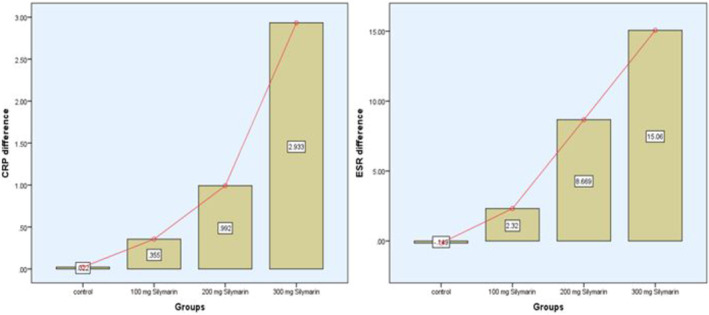
Difference of inflammatory markers of all group participants between baseline and after 3 months.

The inflammatory indicators in individuals with NAFLD are thoroughly examined in Table 5 at baseline and 3 months after taking cookies enhanced with silymarin at different dosages (low: 200 mg, medium: 300 mg, and high: 400 mg). C‐reactive protein (CRP) levels varied little in the placebo group, with mean values. The greatest concentration of silymarin, Treatment 3, on the other hand, showed a significant decrease in CRP levels from 6.32 to 3.39 mg/L (*p*‐value = .003), indicating a possible anti‐inflammatory action. A significant drop in CRP levels was also seen in Treatment 2, highlighting the potential advantages of silymarin in reducing inflammation linked to NAFLD. All treatment groups demonstrated a statistically significant drop in erythrocyte sedimentation rate (ESR) values; Treatment 3 exhibited the greatest reduction, going from 38.72 to 23.86 mm/h (*p*‐value = .000). A possible abatement of systemic inflammation with increased concentrations of silymarin is indicated by this significant decrease in ESR levels. Figure 4 shows the difference between baseline and after 3 months of the study.

**TABLE 5:** Inflammatory markers of all group participants with NAFLD at baseline and after 3 months.

Groups	Parameters			
	CRP (mg/L)	CRP (mg/L)	ESR (mm/h)	ESR (mm/h)
	Before	After	Before	After
Placebo				
Mean ± SD	6.13 ± 0.37	6.11 ± 0.45	37.53 ± 2.05	37.68 ± 2.12
Median (Min–Max)	5.97 (5.84–7.01)	5.96 (5.55–7.12)	37.04 (34.97–41.07)	37.32 (34.97–41.79)
Treatment 1				
Mean ± SD	6.34 ± 0.47	5.98 ± 0.49	37.93 ± 1.22	35.61 ± 2.39
Median (Min–Max)	6.06 (5.86–7.09)	5.93 (5.33–6.71)	38.19 (35.62–40.29)	36.92 (31.76–38.44)
Treatment 2				
Mean ± SD	6.35 ± 0.47	5.36 ± 0.79	38.57 ± 1.71	29.90 ± 5.62
Median (Min–Max)	6.16 (5.87–7.07)	5.61 (3.89–6.90)	38.85 (34.04–41.03)	30.01 (22.34–38.98)
Treatment 3				
Mean ± SD	6.32 ± 0.38	3.39 ± 1.68	38.72 ± 1.62	23.65 ± 4.87
Median (Min–Max)	6.11 (5.96–7.00)	3.85 (1.03–5.96)	38.74 (35.23–40.99)	23.74 (18.43–39.26)
*p*‐value	.277^a^	.000^b^	.167^b^	.000^a^

*Note*: Placebo‐ simple cookies, T^1^‐2 (1 morning and 1 evening), T^2^‐3 (1 ½ in morning and 1 ½ in evening), T^4^‐4 (2 in morning and 2 in evening).

Abbreviations: CRP, C‐reactive protein; ESR, erythrocyte sedimentation rate; NAFLD, nonalcoholic fatty liver disease.

^a^ Kruskal–Wallis test.

^b^ One‐way ANOVA.

Analysis of variance (ANOVA) and Kruskal–Wallis tests are among the statistical analyses that support the observed differences and show that Treatment 3 is the most successful in lowering CRP and ESR levels. These results highlight the possible anti‐inflammatory properties of cookies containing silymarin, indicating their potential use in managing inflammation linked to NAFLD.

A detailed examination of renal function tests (RFTs) conducted on individuals with NAFLD at baseline and 3 months after taking cookies enhanced with silymarin at different dosages (low: 200 mg, medium: 300 mg, and high: 400 mg) is given in Table 6.

**TABLE 6:** Renal function tests of all group participants with NAFLD at baseline and after 3 months.

Groups	Parameters (mg/dL)									
	Albumin	Albumin	Total protein	Total protein	BU	BU	BUN	BUN	Creatinine	Creatinine
	Before	After	Before	After	Before	After	Before	After	Before	After
Placebo										
Mean ± SD	6.29 ± 0.61	6.09 ± 0.42	9.51 ± 0.94	8.89 ± 0.63	25.84 ± 1.38	24.78 ± 1.87	33.46 ± 2.34	31.76 ± 2.68	0.92 ± 0.06	0.90 ± 0.05
Median (Min–Max)	5.95 (5.84–7.84)	5.90 (5.84–6.96)	9.41 (7.91–11.84)	8.90 (7.67–9.91)	25.73 (22.65–28.37)	24.21 (22.44–28.46)	33.52 (29.29–37.27)	31.51 (28.07–36.17)	0.91 (0.84–1.08)	0.90 (0.84–1.03)
Treatment 1										
Mean ± SD	6.43 ± 0.60	5.75 ± 0.33	9.50 ± 0.89	9.01 ± 0.65	25.89 ± 1.02	24.00 ± 1.24	34.96 ± 2.59	31.47 ± 3.24	0.93 ± 0.06	0.87 ± 0.03
Median (Min–Max)	6.04 (5.86–7.89)	5.86 (4.89–5.95)	9.38 (7.89–10.95)	8.89 (7.67–9.92)	25.76 (24.38–27.75)	23.96 (22.34–27.03)	34.15 (31.60–40.26)	31.92 (27.07–38.89)	0.91 (0.86–1.06)	0.88 (0.82–0.95)
Treatment 2										
Mean ± SD	6.32 ± 0.44	5.29 ± 0.52	9.55 ± 0.95	8.16 ± 0.76	25.71 ± 1.014	23.21 ± 1.35	35.89 ± 2.25	28.81 ± 2.53	0.94 ± 0.07	0.85 ± 0.04
Median (Min–Max)	6.05 (5.92–6.92)	4.89 (4.83–5.97)	9.85 (7.90–11.89)	7.96 (6.07–9.18)	25.76 (24.08–27.73)	23.14 (20.96–25.52)	36.19 (32.26–38.98)	28.47 (23.09–33.58)	0.92 (0.85–1.10)	0.85 (0.83–0.97)
Treatment 3										
Mean ± SD	6.48 ± 0.46	4.45 ± 0.64	9.33 ± 1.07	7.12 ± 0.69	25.80 ± 1.143	21.18 ± 1.97	35.54 ± 2.12	24.82 ± 3.37	0.96 ± 0.07	0.88 ± 0.04
Median (Min–Max)	6.40 (5.96–7.03)	4.39 (3.84–5.95)	9.62 (7.61–11.55)	7.04 (6.07–7.97)	25.56 (24.48–27.84)	20.45 (18.07–24.43)	35.56 (31.80–40.29)	23.99 (21.45–34.89)	0.98 (0.85–1.10)	0.88 (0.83–0.99)
*p*‐value	.112^a^	.000^a^	.449^a^	.000^a^	.977^b^	.000^b^	.24^b^	.000^b^	.305^b^	.015^b^

*Note*: Placebo‐ simple cookies, T^1^‐2 (1 morning and 1 evening), T^2^‐3 (1 ½ in morning and 1 ½ in evening), T^4^‐4 (2 in morning and 2 in evening).

Abbreviations: BU, blood urea; BUN, blood urea nitrogen; NAFLD, nonalcoholic fatty liver disease.

^a^ Kruskal–Wallis test.

^b^ One‐way ANOVA.

**FIGURE 5:**
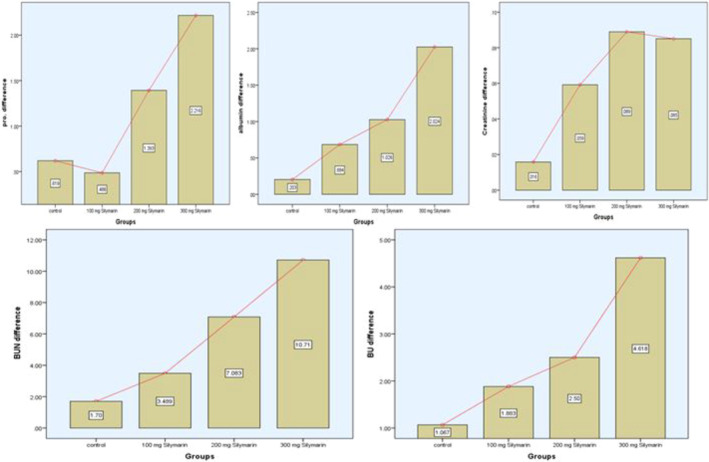
Renal function tests (RFTs) difference of all group participants between baseline and after 3 months.

Renal parameters showed only minor changes in the placebo group: albumin, total protein, blood urea (BU), blood urea nitrogen (BUN), and creatinine levels were all slightly lower. Notably, several renal indicators showed notable improvements in Treatment 3, which had the greatest quantity of silymarin. In particular, total protein levels significantly decreased from 9.33 to 7.12 g/dL (*p*‐value = .001), while albumin levels also decreased from 6.48 to 4.45 g/L (*p*‐value = .001). Significant drops in BU and BUN levels were also seen, highlighting the possible renoprotective benefits of increased silymarin concentrations. The important kidney function marker, creatinine, dropped dramatically from 0.96 to 0.88 mg/dL (*p*‐value = .007), indicating a beneficial effect on renal health. Regarding all kidney indicators, Treatment 3 consistently had the best outcomes. Figure 5 shows the difference between baseline and after 3 months of the study. ANOVA and Kruskal–Wallis tests are among the statistical analyses that support the observed differences and show that Treatment 3 is the most successful in enhancing renal function. These results highlight the possible renoprotective benefits of cookies enriched with silymarin, particularly at higher dosages.

A comprehensive examination of lipid profile changes (Table 7), specifically concerning treatment for NAFLD, indicates encouraging results when different dosages of silymarin supplements are taken. Significant changes in important parameters, such as total cholesterol (TC), high‐density lipoprotein (HDL), low‐density lipoprotein (LDL), and triglycerides (TG), are revealed by the thorough examination of lipid profile changes in response to various treatments. The research investigated the differences between the pretreatment (Before) and posttreatment (After) levels in four groups: Placebo, Treatment 1, Treatment 2, and Treatment 3.

**TABLE 7:** Lipid profile of all group participants with NAFLD at baseline and after 3 months.

Groups	Parameters (mg/dL)							
	TC	TC	HDL	HDL	LDL	LDL	TG	TG
	Before	After	Before	After	Before	After	Before	After
Placebo								
Mean ± SD	207.32 ± 1.31	206.61 ± 2.84	42.15 ± 4.31	43.20 ± 4.75	152.36 ± 9.10	151.19 ± 9.75	178.31 ± 6.62	175.67 ± 6.59
Median (Min–Max)	207.56 (205.15–209.02)	207.08 (200.60–210.37)	42.12 (36.25–47.74)	43.00 (37.02–49.24)	156.04 (129.98–160.22)	155.11 (127.87–158.90)	180.78 (165.35–188.35)	178.30 (158.55–181.44)
Treatment 1								
Mean ± SD	206.59 ± 1.50	205.38 ± 1.90	41.33 ± 3.44	46.17 ± 4.97	154.11 ± 5.02	150.37 ± 5.48	179.81 ± 5.19	173.41 ± 6.83
Median (Min–Max)	206.48 (203.55–208.65)	205.89 (201.08–207.40)	40.57 (37.59–49.42)	45.08 (38.94–56.61)	155.58 (143.76–159.91)	151.36 (141.31–159.48)	181.34 (168.65–185.75)	177.00 (159.05–179.97)
Treatment 2								
Mean ± SD	207.53 ± 1.49	200.42 ± 3.30	41.72 ± 3.46	48.50 ± 5.13	151.45 ± 7.58	146.04 ± 7.68	179.53 ± 5.60	171.54 ± 7.55
Median (Min–Max)	207.93 (205.48–210.00)	200.00 (196.50–206.48)	40.90 (38.21–49.95)	47.63 (43.47–59.68)	152.36 (132.00–160.22)	148.55 (125.30–157.67)	181.39 (167.23–186.35)	175.04 (155.05–178.97)
Treatment 3								
Mean ± SD	206.88 ± 2.52	199.40 ± 2.23	42.42 ± 3.39	58.87 ± 3.20	151.74 ± 8.11	142.61 ± 7.99	179.56 ± 6.60	158.40 ± 12.81
Median (Min–Max)	207.45 (201.10–210.10)	198.83 (197.30–205.48)	41.40 (38.71–49.86)	59.94 (49.95–62.15)	155.27 (132.00–160.22)	141.50 (125.30–152.41)	181.00 (168.40–188.38)	151.97 (145.99–188.85)
*p*‐value	.348^b^	.000^a^	.632^a^	.001^a^	.806^a^	.010^b^	.897^b^	.000^a^

*Note*: Placebo‐ simple cookies, T^1^‐2 (1 morning and 1 evening), T^2^‐3 (1 ½ in morning and 1 ½ in evening), T^4^‐4 (2 in morning and 2 in evening).

Abbreviations: BU, blood urea; BUN, blood urea nitrogen; HDL, high‐density lipoprotein; LDL, low‐density lipoprotein; NAFLD, nonalcoholic fatty liver disease; TC, total cholesterol; TG, triglycerides.

^a^ Kruskal–Wallis test.

^b^ One‐way ANOVA.

**FIGURE 6:**
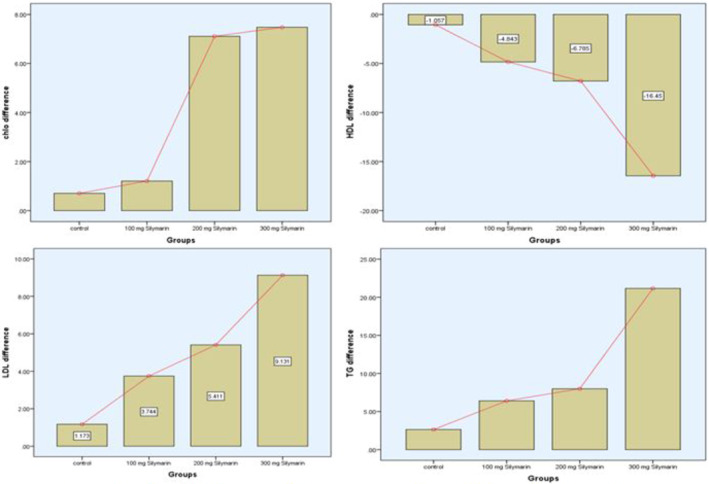
Lipid profile difference of all group participants between baseline and after 3 months.

High‐density lipoprotein (HDL) levels show a noteworthy improvement, HDL levels significantly increased with Treatment 3, rising from 42.42 to 58.87 mg/dL. This suggests that supplementing with silymarin, particularly at higher doses (400 mg), may be essential for raising HDL levels. Increased HDL is linked to better liver function and a lower chance of cardiovascular diseases, two important factors to take into account while dealing with NAFLD. The investigation also shows that all groups’ posttreatment levels of LDL were significantly lower (*p*‐value = .006). The greatest impact is shown by Treatment 2, which has a higher concentration of silymarin and causes LDL levels to drop from 151.45 to 146.04 mg/dL. Reduced LDL levels often correspond to a lower risk of atherosclerosis, indicating a possible function for silymarin in reducing the cardiovascular risks related to NAFLD. Additionally, the study finds that there have been statistically significant improvements in the levels of triglycerides (TG) (*p*‐value = .001). Treatment 3, which has a higher concentration of silymarin, has demonstrated the greatest reduction in TG, from 179.56 to 158.40 mg/dL. Since NAFLD is frequently connected to elevated triglycerides, the observed reductions in TG levels raise the possibility that silymarin may have a therapeutic benefit in reducing lipid abnormalities associated with liver disorders. Figure 6 shows the difference between baseline and after 3 months of the study.

The study's findings highlight the potential advantages of silymarin supplementation, especially at higher dosages, in terms of modifying lipid profiles during the duration of treating NAFLD. Treatment 3 is particularly promising, with significant decreases in TG, LDL, and HDL values. In order to confirm the noted modifications and establish silymarin supplementation as a viable supplementary treatment for NAFLD that addresses the disease's cardiovascular and liver‐related components, these results emphasize the significance of additional studies and clinical trials.

## 4 DISCUSSION

This randomized controlled clinical study was conducted to assess the efficacy of different dosages of silymarin‐supplemented cookies two times daily for 3 months on liver aminotransferases, inflammatory markers, lipid profile, and renal function tests in individuals with NAFLD without any specific adverse effects. The study demonstrated a notable decrease in inflammatory markers and liver enzyme levels among individuals receiving silymarin supplementation. Also, this clinical research is the first to examine in humans the impact of cookies supplemented with silymarin on liver enzymes' and antioxidants' lipid profile and renal function tests in NAFLD patients. Importantly, the placebo group did not exhibit a similar reduction in aminotransferase levels after 3 months, highlighting that the observed variations were likely attributed to the specific therapy administered. The above findings suggest a potential hepatoprotective effect of silymarin, as evidenced by the significant improvement in liver enzyme profiles, emphasizing its role in ameliorating liver function in the context of NAFLD. The results revealed several important findings that permit discussion.

Silymarin has a number of advantageous qualities that have been discovered, including the modulation of insulin resistance, antioxidant, hepatoprotective, anti‐inflammatory, and antifibrotic activities (Gillessen & Schmidt, 2020). According to our results, the findings of three clinical studies indicated the protective effects of silymarin against liver enzymes, 74.4% and 65.5% reductions in ALT and AST, respectively (Ahmed et al., 2022); 67.7% and 63.1% reductions in ALT and AST, respectively (Mirzaei et al., 2021), and 62.3% and 61.5% reductions in ALT and AST, respectively (Jamalian et al., 2020). Another randomized experiment assessing silymarin's impact on biomarkers of the hepatic inflammatory process, such as ALT and AST, in individuals with acute clinical hepatitis of any origin revealed no discernible change. On the other hand, silymarin markedly reduced subjective biliary retention symptoms and indicators (El‐Kamary et al., 2009).

Nonalcoholic fatty liver disease (NAFLD) is a prevalent liver disorder affecting adults and children, with increasing incidence among children and adolescents due to rising obesity rates. It is a major cause of liver cirrhosis and is part of the metabolic syndrome, which includes obesity, hyperinsulinism, glucose intolerance, and hyperlipidemia (Dongiovanni et al., 2021). Treatment for NAFLD mainly involves a restricted diet, quitting harmful substances, and treating metabolic conditions like diabetes and hyperlipidemia. Despite variable outcomes, numerous studies have investigated the role of dietary modifications, physical activity, and certain drugs in managing NAFLD (Fernández et al., 2022). This might be explained by the availability of milk thistle seeds, which have silymarin in them. Silymarin may help NAFLD patients experience a significant drop in their blood levels of the ALT and AST enzymes (Shaikh et al., 2021).

Fifty NAFLD patients participated in a randomized clinical trial that proved silymarin's effectiveness. The patients' mean ALT and AST values dropped from 103.1 to 41.4 U/L to 53.7–29.1 IU/mL, respectively, after receiving one 140 μg of silymarin pill daily for 2 months (*p* < .001) (Atarodi et al., 2022). The impact of silymarin on variations in transaminase levels was also validated by another investigation. While it should be emphasized that baseline transaminase levels were within the normal range, these results are in contrast to the current findings, which did not show any significant increases in transaminase levels. The effectiveness of silymarin for treating nonalcoholic steatohepatitis was investigated by Solhi et al. (2014). The study's findings indicated that patients taking silymarin had a significant reduction in their liver enzyme levels (Solhi et al., 2014). The effects of silymarin on serum levels of aminotransferases in patients with nonalcoholic steatohepatitis were studied by Masoodi et al. (2013). The findings of the study demonstrated that silymarin administration could effectively reduce hepatic aminotransferases without affecting the patients’ BMI. *Silibum marinum*'s antioxidant properties, which can shield the liver from toxins, appear to be the cause of the drop in AST and ALT following silymarin ingestion. Mirnezami et al.'s study's findings demonstrated that silymarin and its nanocrystals reduced the diameter of the invasive hepatocyte nucleus in intoxicated rats as well as the elevated liver inhibitory enzyme brought on by ingestion of titanium dioxide nanoparticles (Mirnezami et al., 2020). The current study's findings are in line with those of the previously stated investigations.

The most recent data in this field have been provided in this paper, as far as we are aware. The impact of silymarin‐supplemented cookies on other liver and blood parameters was not assessed since laboratory testing is expensive, which is one of the study's major weaknesses. It is recommended that future research measure all blood and liver parameters. It is advised to look into the impact of cookies enriched with silymarin on patients, as all of the participants in the current study had NAFLD. Additionally, given that individuals with NAFLD have elevated liver enzyme levels, it is advised to look at the impact of cookies on lipid profiles, inflammatory markers, and different renal function tests.

## 5 CONCLUSION

The study investigates the potential of silymarin‐supplemented cookies in managing NAFLD, a public health concern characterized by fat accumulation and severe disorders like NASH. A clinical trial in Lahore, Pakistan, involved 64 NAFLD patients. After 3 months, the treatment groups showed significant decreases in liver enzymes, ALT and AST levels, ALP levels, and the AST/ALT ratio. Additionally, the intervention reduced inflammatory markers, CRP, and ESR, suggesting potential advantages for liver health.

## AUTHOR CONTRIBUTIONS

**Hafiza Madiha Jaffar:** Conceptualization (lead); data curation (equal); formal analysis (equal); investigation (equal); methodology (lead); resources (lead); software (lead); validation (lead); visualization (lead); writing – original draft (lead); writing – review and editing (lead). **Huma Bader ul Ain:** Conceptualization (equal); methodology (equal); supervision (lead); writing – original draft (equal). **Tabussam Tufail:** Conceptualization (equal); supervision (equal); writing – review and editing (equal). **Asif Hanif:** Formal analysis (equal); software (lead); writing – review and editing (supporting). **Tabarak Malik:** Writing – review and editing (equal).

## CONFLICT OF INTEREST STATEMENT

The authors declare no conflicts of interest.

## ETHICS STATEMENT

This study was approved by the Institutional Review Board (IRB) of the University of Lahore, Pakistan.

## INFORMED CONSENT

Written informed consent was obtained from all study participants.

## Data Availability

Data are contained within the article.
